# Efficacy of the DRL orthokeratology lens in slowing axial elongation in French children

**DOI:** 10.3389/fmed.2023.1323851

**Published:** 2024-01-04

**Authors:** António Queirós, Pauline Rolland le Moal, Karine Angioi-Duprez, Jean-Paul Berrod, Jean-Baptiste Conart, Aurélia Chaume, Jaume Pauné

**Affiliations:** ^1^Clinical and Experimental Optometry Research Lab (CEORLab), School of Science University of Minho, Braga, Portugal; ^2^Physics Center of Minho and Porto Universities, Braga, Portugal; ^3^Department of Ophthalmology, University Hospital of Nancy, Vandoeuvre-lès-Nancy, France; ^4^Private Ophthalmogoly Clinic, Laxou, France; ^5^Teknon Medical Center, Barcelona, Spain; ^6^Faculty of Optics and Optometry Polytechnic, University of Catalonia, Terrassa, Spain; ^7^Optometry School Optometry, University of Montreal, Montreal, QC, Canada

**Keywords:** orthokeratology, myopia control, axial length, refractive errors, contact lens

## Abstract

**Background:**

This study aims to assess and compare the impact of Orthokeratology Double Reservoir Lens (DRL) versus Single Vision Lenses (SVL) on axial elongation and anterior chamber biometric parameters in myopic children over a 6- and 12-month treatment period in France.

**Methods:**

A retrospective study involving 48 patients aged 7 to 17 years, who underwent either orthokeratology treatment or single-vision spectacle correction, was conducted. Changes in refractive error, axial length, and anterior chamber depth were examined.

**Results:**

Twenty-five patients comprised the Orthokeratology (OK) group, while twenty-three were in the control group (single-vision spectacle group). Significant increases in mean axial length were observed over time in both the control (0.12 ± 0.13 mm and 0.20 ± 0.17 mm after 6 and 12 months, respectively; *F* (2,28.9) = 27.68, *p* < 0.001) and OK groups (0.02 ± 0.07 mm and 0.06 ± 0.13 mm after 6 and 12 months, respectively; *F* (2,29.1) = 5.30, *p* = 0.023). No statistically significant differences in axial length were found between male and female children (*p* > 0.620). Age-specific analysis revealed no significant axial elongation after 12 months in the 14–17 years group in the OK group. Anterior biometric data analysis at 6 and 12 months showed statistical significance only for the DRL group.

**Conclusion:**

Orthokeratology resulted in an 86 and 70% reduction in axial elongation after 6 and 12 months of lens wear, respectively, compared to the single-vision spectacles group. Myopia progression was more pronounced in younger children, underscoring the importance of initiating myopia control strategies at early ages.

## Introduction

Several studies have reported an increase in both the percentage of prevalence of myopia and the average values of refractive error in recent years (1). This trend is particularly pronounced in Asian countries, but also reflected in North America and Europe (2–4). Forecasts estimate that half the world’s population will be myopic by 2050, the prevalence of myopia is increasing in Europe. Recent publications have showed age-standardized myopia prevalence for those completing primary, secondary, and higher education was 25.4, 29.1, and 36.6% (5). In France, a study of involving over 100,429 individuals, performed in 4 different eye treatment centers, showed prevalences of mild, moderate, high, and very high myopia of 25.1, 10.6, 3.4, and 0.5%, respectively (6). Which in socio-economic terms will have several implications with the pathological problems associated with myopia (7). Axial elongation of the eyeball in high myopia is indeed associated with a higher risk of pathologic ocular changes including cataract, glaucoma, retinal detachment and myopic maculopathy, bringing increased healthcare costs and ocular comorbidities (8). Therefore, the prevention of myopia onset and axial length progression is of utmost importance (9).

In the last two decades, in response to the myopia “epidemic,” several treatments and strategies including optical methods (10–15), pharmaceutical (16, 17) interventions or even combination of both (18), have been developed in order to halt the progression of myopia.

Nowadays, orthokeratology (OK) is one of the most widely used methods in the world for myopia control. Myopic OK is a technique that uses specially reverse geometry rigid contact lenses designed to flatten the central cornea during overnight wear to eliminate daytime myopia (19, 20). Several studies have shown its effectiveness: revealing a 32 to 63% reduction in axial elongation in children wearing OK compared to those wearing only conventional glasses correction (12, 21–25).

However, there is a great individual variability in response to OK treatment, caused by many factors such as age of onset, initial refractive error, corneal power change, pupil diameter and heredity (21, 24, 26–28). The majority of OK studies have been conducted in Asia and in Asian populations, and it is increasingly important to understand the impact of this treatment on Caucasian children and the potential benefits of this approach. Recently, studies with various designs of orthokeratology contact lenses, as well as, with different designs in the geometry of the posterior surface have been done showing a great effectiveness in controlling the progression of myopia with this type of optical devices. Recently Zang et al. evaluated Chinese children aged 8–13 years, showing progression of myopia varied depending on the type of orthokeratology lens design. They used 3 different types of optical zones in orthokeratology contact lenses and showed that smaller and more aspherical treatment zones may be beneficial for reducing axial increment in children with this treatment, regardless of the myopic refraction value at the start of treatment (29). Similar results were obtained by Li et al. under equal design and age conditions. They obtained an increase in control of myopia progression about 50% higher in orthokeratology contact lenses with a posterior optical zone diameter of 5 mm (AL = 0.13 ± 0.13 mm) compared to 6 mm (AL = 0.28 ± 0.22 mm) (30). The results of the previous studies are in agreement with a 24-month longitudinal study of French children aged 5 to 17 years with the Double Reservoir Lens (DRL) orthokeratology contact lens, where the authors showed a 77 and 80% rate of control of myopia progression, at 12 months and 24 months, respectively, when compared to spectacles (31).

Considering the impact of OK as an effective method to control myopia progression, the aim of this retrospective study is to assess and compare the evolution of refractive error, axial length, and anterior chamber biometric parameters (Central corneal thickness, Anterior Chamber Depth and Lens Thickness Depth) over a period of 12 months in French children aged 7 to 17 years treated with orthokeratology or monofocal vision correction spectacles.

## Methods

### Patients and study design

A retrospective review of medical records was performed on 48 consecutive myopic children who underwent OK treatment or single-vision spectacle correction at a private ophthalmology clinic in Nancy from November 2019 to December 2020. The treatment modality was chosen by the patients and their parents themselves after receiving complete information on the risks and benefits of each method. All parents of the participants provided an informed consent, which was associated with the medical record. The study was conducted in accordance with the tenets of the Declaration of Helsinki and was approved by the Ethics committee of the French Society of Ophthalmology.

Consecutive 48 myopic patients were selected according to the following inclusion criteria (7 to 17 years old; Myopia between 0.50 D and 7.00 D; Astigmatism ≤4.00 D with-the-rule 180 ± 20°; Anisometropia ≤1.50 D; Best-corrected visual acuity (BCVA) ≥ 20/20; Follow-up period >12 months) and exclusion criteria (Prior history of any other myopia control treatment - except for single vision distance spectacles; Contraindication for contact lens wear or orthokeratology; Preexisting ocular – amblyopia, strabismus, ocular inflammation, trauma, or surgery - or systemic disease; Poor compliance to lens wear, examination or follow-up). Patients were divided into 2 groups based on the treatment modality: the OK group consisting of patients who used OK lenses for myopia correction and the control group who preferred single-vision spectacles to correct their myopia. All patients underwent a detailed ophthalmologic examination by the same examiner at baseline, 6 months and 12 months. The examination included assessment of best-corrected visual acuity (BCVA) using projected-light Snellen charts, non-cycloplegic manifest refraction, ocular biometric measurements, biomicroscopy with anterior segment evaluation and fundus imaging using the Cirrus-HD OCT (Carl Zeiss Meditec, Inc., Dublin, CA, United States).

### Orthokeratology lenses

Patients were fitted with a Double Reservoir Lens (DRL) (Precilens, Creteil, France) according to the manufacturer’s instructions based on topographic values, refraction and corneal diameter. DRL lenses are made of hexafocon A material with an oxygen permeability of 100 ISO/Fatt, refractive index of 1.415 and wetting angle of 49 degrees measured with the captive bubble method.

After lens dispensing, patients were instructed to wear their OK lenses every night for at least six consecutive hours. They were required to return for routine OK aftercare visits at 1 day, 10 days, 1 and 3 months post-lens delivery. At each visit, a slit-lamp examination was performed to check for OK lens related complications and any adverse events. Additional visits were provided when needed.

### Refraction and ocular biometrics measurements

All participants underwent cycloplegic objective refraction before entering the study. Autorefraction and keratometry were performed using an AutoKerato-Refractometer (TONOREF III, NIDEK, Japan). Noncycloplegic manifest refraction was assessed by an experienced specialist at the baseline, 6-month and 12-month visits. In the case of orthokeratology group, a refraction after stabilization (1 month) was performed and considered as the refractive baseline parameter for this group. The change at 6 and 12 month was considered as the difference between refraction at these follow-up and baseline at 1 month time OK wear. Since participants were with-the-rule (WTR) astigmatism, axis at 0° ± 30°, refractive parameters were not converted to power vectors (M, J0, J45).

Ocular biometrics including central corneal thickness (CCT), anterior chamber depth (ACD), lens thickness (LT), anterior segment length (ASL) (which is the sum of CCT, ACD and LT) and AL [distance between the anterior cornea and the retinal pigment epithelium (RPE)] were measured using a noncontact optic biometric device (Lenstar LS 900; Haag Streit AG, Koeniz, Switzerland). Five successive measurements were taken from each subject at each measurement session and a mean was obtained. All measurements were carried out between 2 and 5 pm to limit the influence of diurnal variations (32).

### Data collection

Baseline characteristics included demographic data, amount of myopia and refractive astigmatism, spherical equivalent error (SER), BCVA, anisometropia, myopic progression over the past 6 months, keratometry, and ocular biometrics parameters.

We collected the following data at baseline, 6 months and 12 months: amount of myopia and refractive astigmatism, SER, BCVA, keratometry and ocular biometrics parameters. We also recorded complications related to the OK lenses during the study period (such as not wearing them every day), but these patients were not included in the analysis of the results. Because similar data were observed at baseline in both eyes, only data from the right eye were analyzed for both groups.

### Statistical analysis

Quantitative parameters were described as mean and standard deviation. The statistical package SPSS v.21 (SPSS Inc., Chicago, IL, United States) was used to conduct the statistical analysis. The Shapiro–Wilk test was applied to evaluate the normality of data distribution. Qualitative parameters were expressed as frequency and percentage. The chi-squared test was used to analyze the variables between the groups gender and age groups. The t-test independent samples or Mann–Whitney U-test were used for the analysis of differences in refraction variables before and after treatment and before and 12 months, respectively, for the normal distribution or not of the variables. Comparison between the three visits was made with the repeated measures ANOVA test with Bonferroni adjustment or Friedman Test for non-parametric variables. The Mauchly’s test was used to check for sphericity. When there was no sphericity, the values of Greenhouse–Geisser or Huynh-Feldt according to the value of Epsilon’s coefficient. The comparison of results is always presented as “Mean ± Standard Deviation” for better visualization of trends. A value of *p* <0.05 was accepted as statistically significant.

## Results

According to the inclusion criteria in this study, 48 eyes from 48 patients were included: 25 eyes were included in the OK group and 23 eyes were included in the control group. “*A posteriori*” sample size calculation predicts that the 17 subjects included in each group warrant enough statistical power to test our hypothesis that differences result in axial length. According to the randomized tests for two means (GPower3.1) and based on the previous research (33), it was estimated to induce at least a 0.10 mm difference in AL between the two groups over 1y, with a standard deviation (SD) of 0.10 mm. A sample size of 17 subjects in each group was required to achieve a power (1-β) of 80% and a significance level (α) of 0.05.

### Baseline characteristic

Baseline characteristics for both groups are presented in Table 1. Fifteen girls (60.0%) and 10 boys were included in the OK group, while nine girls (39%) and 14 boys in the control group (*p* = 0.149). The mean age of patients was 12.24 ± 2.49 and 12.39 ± 3.16 years in the OK and control groups, respectively (*p* = 0.854). There was also no significant difference in age distribution (7–13 years and 14–17 years) between the two groups.

**Table 1:** Demographic data of quantitative variables at the beginning of treatment.

	DRL (*n* = 25)	Glasses (*n* = 23)	diff	*p*
Age (years)	12.24 ± 2.49	12.39 ± 3.16	−0.15 ± 0.82	0.854§
Sphere (D)	−2.57 ± 1.23	−2.32 ± 1.42	−0.25 ± 0.38	0.207¥
Cylinder (D)	−0.57 ± 0.51	−0.65 ± 0.67	0.08 ± 0.17	0.916¥
Spherical Equivalent (D)	−2.86 ± 1.27	−2.64 ± 1.50	−0.21 ± 0.40	0.252¥
Axial Length (mm)	24.68 ± 0.97	24.67 ± 1.05	0.01 ± 0.29	0.968§
Central corneal thickness (μm)	549 ± 38	547 ± 34	2 ± 10	0.797§
Anterior Chamber Depth (mm)	3.34 ± 0.22	3.31 ± 0.22	0.03 ± 0.06	0.673§
Lens Thickness (mm)	3.40 ± 0.16	3.42 ± 0.14	−0.01 ± 0.04	0.767§
*Gender*				
Female	15	9	24	0.149*
Male	10	14	24	
*Age group*				
7–13 years	18	13	31	0.263*
14–17 years	7	10	17	

¥ *t*-test independent samples, § U de Mann–Whitney, * chi-squared test.

The overall analysis of the data at the start of treatment show that there were no statistically significant differences between the data collected for the groups under study (DRL and Glasses). The difference in refractive error M between subjects assigned to the DRL and Glasses groups was 0.21 ± 0.40D greater in the DRL group (*p* = 0.252, t-test independent samples). Although there was a relatively smaller difference between them in axial length (0.01 ± 0.29 mm, *p* = 0.968 U de Mann–Whitney), the DRL group had slightly higher values. It is worth noting that the fact that with the standardization of the sample (25/23 in each group) the differences between the groups under study, for all variables, are less than 20% of the standard deviation, with the smallest being AL at 1%.

### Refractive error at 6 and 12 months

As shown in Table 2, after 12 months of treatment there was a decrease in the spherical refractive error for the subjects with DRL of +0.02 ± 0.10D (*p* = 0.220) and an increase of-0.52 ± 0.49D (*p* < 0.001) for the subjects with glasses. However, due to the increase in astigmatism in patients with DRL (p < 0.001), significant differences were observed in the spherical equivalent for both treatments after 12 months (*p* < 0.042). A detailed analysis of the spherical equivalent shows that after 1 year of treatment with DRL lenses, the increase in M value was-0.06 ± 0.14D (*p* = 0.042) compared to glasses group-0.57 ± 0.57D (*p* < 0.001). After 1 year of study, pairwise analysis also evidenced that differences only occurred between 6 months and 12 months in the case of the DRL treatment. Table 2 thus allows us to conclude that the control rate of myopia progression in spherical equivalent was 88% in DRL group compared to glasses after 12 months. With a differential increment of 0.50D in the spherical equivalent of spectacle wearers compared to the DRL treatment.

**Table 2:** Refraction data from the longitudinal study at 6 and 12 months of treatment in the comparison between DRL and glasses at Visit 1 (Baseline), 2 (6 months) and 3 (12 Months).

		Baseline	6 months	12 months	Significance	Pairwise Bonferroni
Sphere (D)	DRL	−2.57 ± 1.23	−2.51 ± 1.26	−2.55 ± 1.31	*F* (2,30.6) = 1.59, *p* = 0.220	x
	Glasses	−2.32 ± 1.42	−2.59 ± 1.56	−2.84 ± 1.57	X^2^ (2) = 31.19, *p* < 0.001	V1-V2; V1-V3; V2-V3
Cylinder (D)	DRL	−0.57 ± 0.51	−0.69 ± 0.66	−0.75 ± 0.68	X^2^ (2) = 15.20, *p* < 0.001	V1-V3
	Glasses	−0.65 ± 0.67	−0.72 ± 0.63	−0.74 ± 0.65	X^2^ (2) = 5.57, *p* < 0.062	x
Spherical Equivalent M (D)	DRL	−2.86 ± 1.27	−2.86 ± 1.30	−2.93 ± 1.37	*F* (2,33.4) = 3.98, *p* = 0.042	V2-V3
	Glasses	−2.64 ± 1.50	−2.95 ± 1.63	−3.21 ± 1.64	X^2^ (2) = 33.46, *p* < 0.001	V1-V2; V1-V3; V2-V3
Difference (mm)		M_6 – M_0		M_12 – M_0		
DRL		0.00 ± 0.10		0.06 ± 0.14		
Glasses		0.31 ± 0.13		0.57 ± 0.57		
Difference		−0.31 ± 0.10		−0.51 ± 0.40		
Significance *		0.001		0.001		
%		100		88		

* Friedman Test repeated measures (X^2^) and repeated measures ANOVA (F), * U Mann–Whitney Test.

### Axial length at 6 and 12 months

The longitudinal analysis of axial length shows that on average the eyes of the subjects grew in both treatment groups (*p* < 0.05, Repeated measures ANOVA). However, in the DRL treatment group, statistically significant differences for AL were observed only between Visit 2 (6 months) and Visit 3 (12 months) while in spectacle wearers significant differences occurred between all visits (Table 3).

**Table 3:** Axial length data from the longitudinal study at baseline, at 6 and 12 months of treatment in the comparison between DRL and glasses.

		Baseline	6 months	12 months	Significance	Pairwise Bonferroni
Axial Length (mm)	DRL	24.68 ± 0.97	24.69 ± 0.95	24.74 ± 0.94	*F* (2,29.1) = 5.30, *p* = 0.023	V2-V3
	Glasses	24.67 ± 1.05	24.79 ± 1.04	24.87 ± 1.05	*F* (2,28.9) = 27.68, *p* < 0.001	V1-V2; V1-V3; V2-V3
Difference (mm)		AL_6 – AL_0		AL_12 – AL_0		
DRL		0.02 ± 0.07		0.06 ± 0.13		
Glasses		0.12 ± 0.13		0.20 ± 0.17		
Difference		0.10 ± 0.03		0.14 ± 0.04		
Significance*		0.001		0.005		
%		86.91		70.54		

Repeated measures ANOVA (F); * U Mann–Whitney Test.

**Figure 1:**
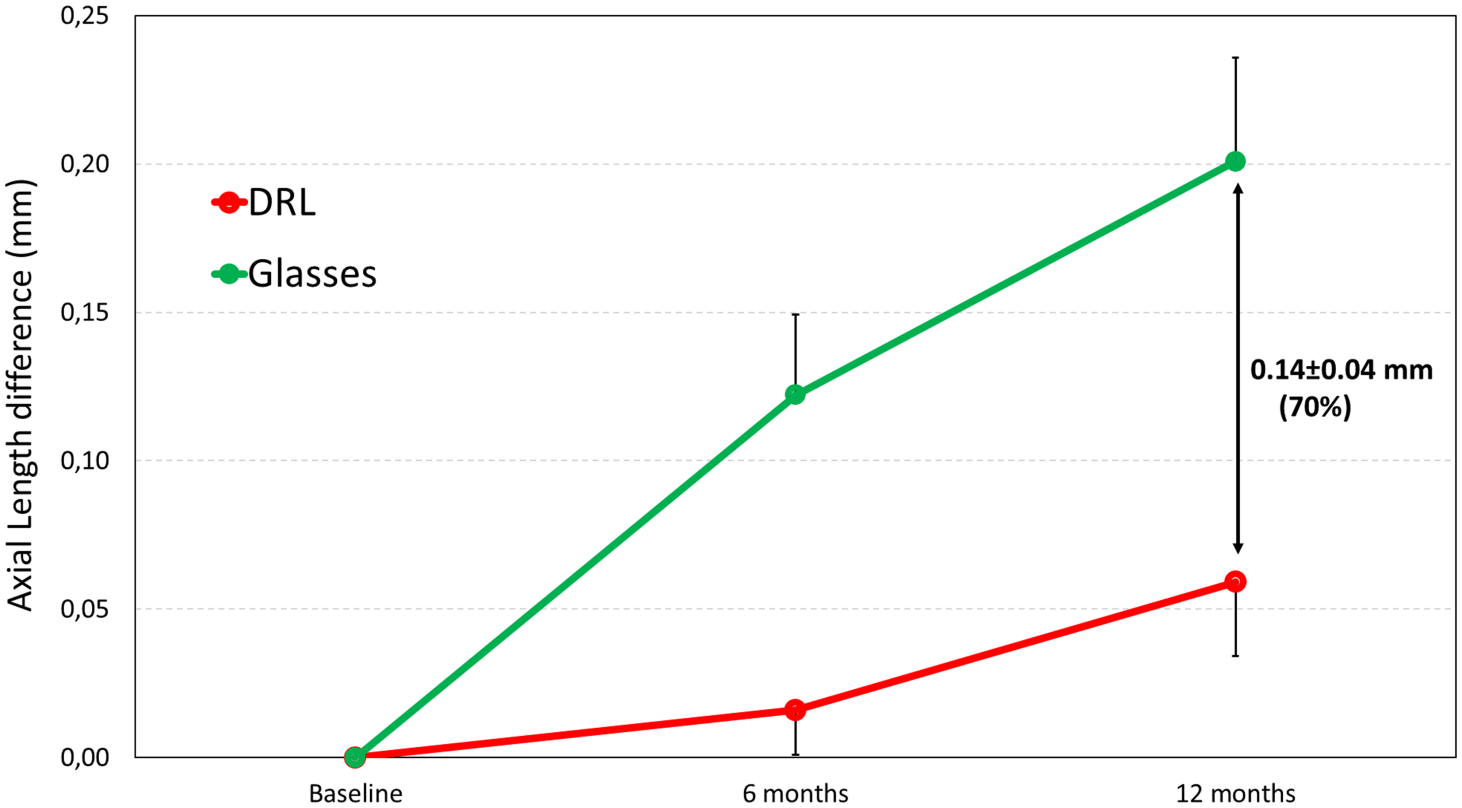
Axial length difference after 6 and 12 months of study for DRL contact lenses and spectacle users.

Figure 1 shows a greater increase in axial length spectacle wearers (0.12 ± 0.13 mm and 0.20 ± 0.17 mm, respectively for 6 and 12 months) compared to the DRL treatment (0.02 ± 0.07 mm and 0.06 ± 0.13 mm, for 6 and 12 months, respectively). This means an effect of DRL treatment in controlling myopia progression by 86% at 6 months and 70% at 12 months (Table 3).

### Anterior biometrics parameters

In the case of glasses wearers there were no significant changes at 6 or 12 months in the anterior biometric parameters of the eye (*p* > 0.182, repeated measures ANOVA). As expected, and according to Table 4, statistically significant differences after DRL contact lens treatment in cornea parameters where the contact lens was applied. These changes increased over time for 6 and 12 month. Table 4 shows the final corneal thickness a decrease (9 μm) as a consequence of the effect of orthokeratology on the anterior surface of the cornea. Nevertheless, the anterior chamber depth (ACD) became smaller by 0.10 mm. It is noteworthy that while in spectacles the lens thickness remains unchanged after 12 months of treatment (*p* = 0.652), in the case of the DRL lenses there was an increase in thickness of 0.11 mm (*p* < 0.001), coupled with the decrease of ACD.

**Table 4:** Anterior biometric data from the longitudinal study at 6 and 12 months of treatment in the comparison between DRL and glasses.

		Baseline	6 months	12 months	Significance	Pairwise Bonferroni
Central corneal thickness (μm)	DRL	549 ± 38	544 ± 38	540 ± 37	X^2^ (2) = 23.05, *p* < 0.001	V1-V3; V2-V3
	Glasses	547 ± 34	546 ± 34	547 ± 36	*F* (2,44.0) = 1.77, *p* = 0.182	X
Anterior chamber depth (mm)	DRL	3.34 ± 0.22	3.26 ± 0.23	3.24 ± 0.24	*F* (2,48.0) = 27.49, *p* < 0.001	V1-V2; V1-V3
	Glasses	3.31 ± 0.22	3.32 ± 0.21	3.32 ± 0.20	*F* (2,23.2) = 0.96, *p* = 0,343	x
Lens thickness (mm)	DRL	3.40 ± 0.16	3.48 ± 0.17	3.51 ± 0.19	*F* (2,34.9) = 36.91, *p* < 0.001	V1-V2; V1-V3
	Glasses	3.42 ± 0.14	3.42 ± 0.14	3.41 ± 0.15	*F* (2,24.6) = 0.25, *p* = 0.652	x

Friedman Test (X^2^) and repeated measures ANOVA (F).

### Axial length by gender

**Figure 2:**
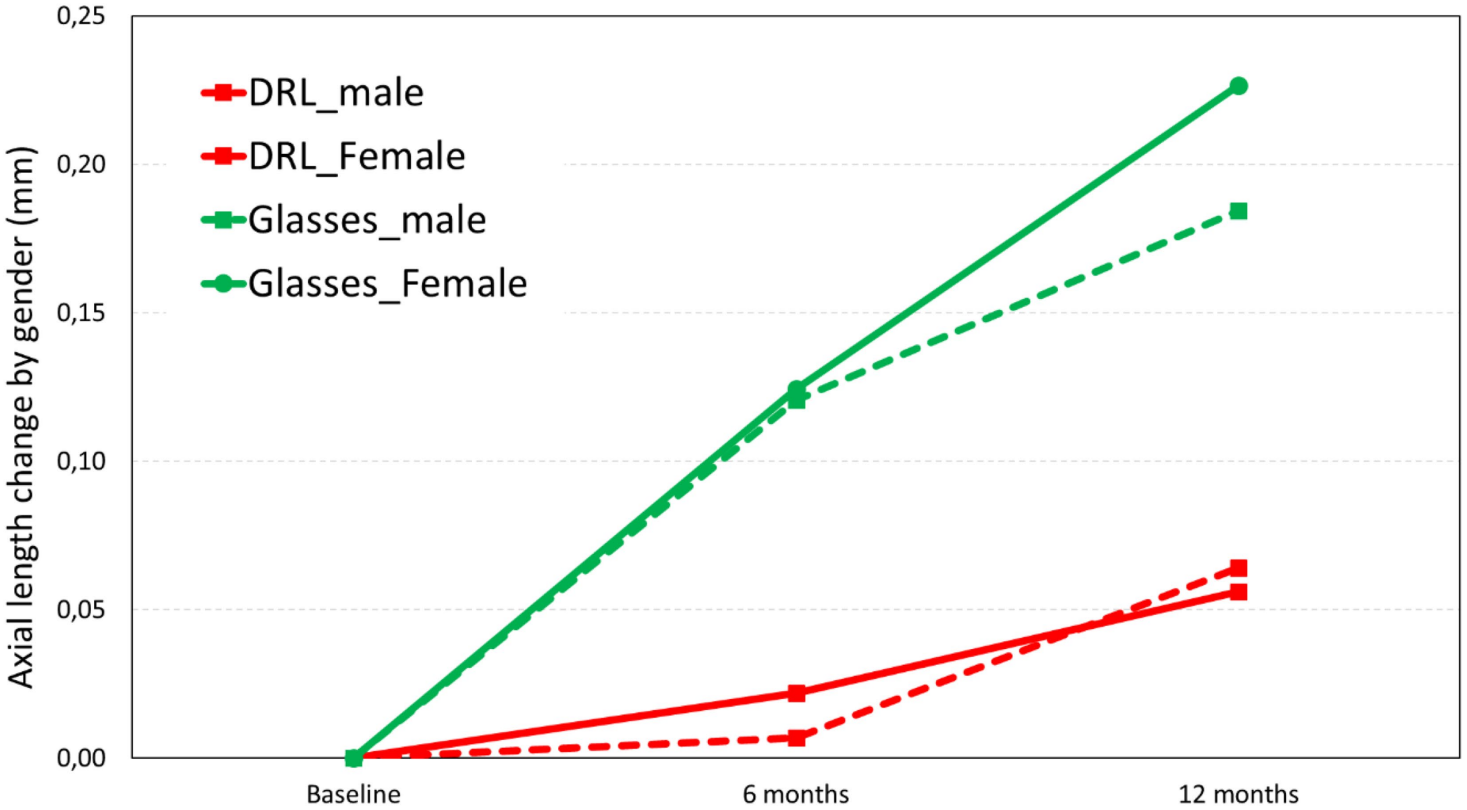
Axial length change in the two treatments (DRL and Glasses) at 6 months and 12 months as a function of gender.

A detailed analysis by gender shows that, on average, there were greater changes in female subjects compared to male subjects at 12 months (Figure 2). However, this pattern is significantly higher in the case of spectacle wearers than with DRL lenses. Male subjects presented an axial length difference between treatments three times higher for spectacles group than DRL lenses (DRL difference = 0.06 mm vs. Glasses difference = 0.18 mm), while in female subjects the increase was approximately four times higher (DRL difference = 0.06 mm vs. Glasses difference = 0.23 mm) at 12 months.

Statistical analysis showed no statistically significant differences in the DRL treatment group over 12 months for males (*p* = 0.200) and females (*p* = 0.071), nor in the comparison between genders over 12 months (*p* = 0.720). The latter result was also true for glasses group (*p* = 0.620, no gender difference over 12 months), although separate analysis between male (*p* = 0.001) and female (*p* = 0.007) showed statistically significant differences over 12 months. Similar results were obtained for 6 months time point, with a 0.01 and 0.02 mm increase in AL for males and females, respectively, and a 0.12 mm increase for male and female Glasses group. These findings suggest a steady increase in axial length for the glasses group over time, while the DRL group exhibited the main axial length increase between 6 to 12 months.

### Axial length by age groups

**Figure 3:**
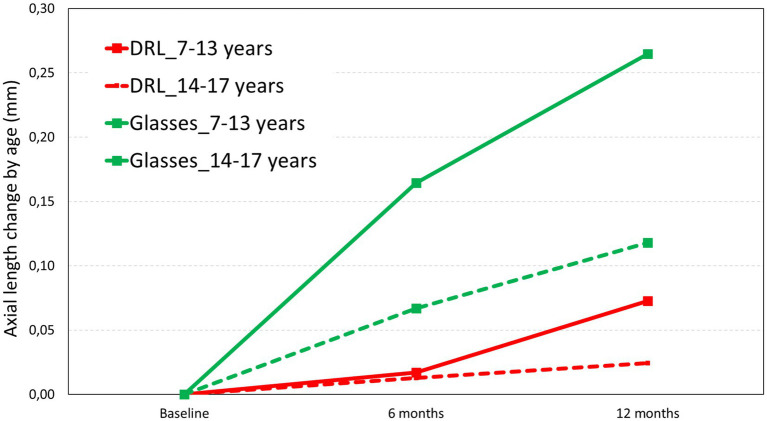
Axial length change in the two treatments (DRL and Glasses) at 6 months and 12 months as a function of age.

As described in the method’s section, the analysis was done separately for lower (ages 7 to 13) and higher (ages 14 to 17) ages. It appears that at lower ages, there were more changes than at higher ages in the glasses treatment. These differences were also observed with higher values in the glasses treatment and lower values with the DRL treatment as can be seen in Figure 3.

The analysis of the data based on age groups revealed statistically significant differences in the 7–13 years age range for both treatments. In the DRL treatment group, it only occurred between 6 months and 12 months (*p* = 0.032, Bonferroni V2-V3) while in glasses treatment group, it occurred between all visits and age groups. However, the analysis between age groups according to the 3 visits showed that for the DRL treatment group there were no statistically differences (*F* (1.2,27.7) = 0.816, *p* = 0.396). In contrast, there were statistically significant differences between age groups, only in the treatment of glasses, as demonstrated by one-way ANOVA, *F* (1.4,29.1) = 4.246, *p* = 0.037.

## Discussion

To the best of our knowledge, this is the first study to investigate the effect of OK treatment on Axial Length changes and anterior chamber biometric parameters, after 6 and 12 months of treatment in a French Caucasian myopic pediatric population. OK was initially developed to eliminate the need for daily optical correction. Many authors have demonstrated that OK is able to correct up to-6.00 D of myopia and-2.50 D of astigmatism (34–36). Our results confirm that OK is effective in correcting daytime myopia and astigmatism, as evidenced by the shift of SER toward plano at 6 and 12 months.

Since the initial evidence in 2005 with LORIC study (11), OK has moved from a refractive option to its use as a mean of slowing myopic progression. As extensively documented in the literature, this study confirms that myopia is likely to progress in children, as demonstrated by the significant increase in AL in both groups. The progression was pronounced in children under 13 in the control group, supporting the importance of early treatment initiation, as mentioned by Cho et al. and Hiraoka et al. (21, 22, 37).

Our results indicate that OK lenses with DRL design is effective in slowing down myopia progression in children, with a Cumulative Absolute Reduction in axial Elongation (CARE) 0.10 ± 0.03, and 0.14 ± 0.04 mm at 6 (*p* = 0.001) and 12 months (*p* = 0.005), respectively, meaning a 86 and 70% of reduction in AL growth compared to single vision glasses in the control group at 6 and 12 months, respectively. This rate compares favorably with the results from previous studies on OK for myopia control which reported 32–63% effectiveness (11, 12, 21, 22, 25, 34, 38, 39). The observed differences may be explained by variations in the design of contact lenses, population, ethnicity and age of OK initiation and follow-up period. Indeed, most studies evaluated the effect of OK on eye growth after 2 to 5 years of OK lens wear (12, 22, 39). In a randomized clinical trial over 2 years, Cho et al. demonstrated a better myopic control in the first 6 months of the study period compared to the subsequent 6-months periods (21). Similarly, Hiraoka et al. (22) noted an apparent reduction in efficacy of OK over time, with no additional beneficial effect after 3 years of lens wear. In fact, this reduction was not due to reduced efficacy of OK but to the slowing of myopic progression in the control group with age, as mentioned by Cho et al. (21). Therefore, it is important to consider the observation period when comparing studies. Our findings align with those of the ROMIO and TO-SEE studies which reported 55–61% effectiveness after 6 months of OK treatment and a mean difference in axial elongation of 0.11 ± 0.01 and 0.12 ± 0.05 mm compared with spectacle-wearing children (21, 34). However, these studies were done in a Chinese population and a ranging in age from 6 to 10 years. In contrast, Santodomingo-Rubido et al. (40) found a lower myopic retardation rate (33%) at 12 months in a Spanish population, with a mean difference in AL growth (CARE) of 0.15 mm between the OK and the control group but with a 0.37 mm/year increase for Single Vision group that is higher that our results. They speculated that Caucasian patients might have a lower propensity to develop and progress in myopia compared to Asians, possibly because of differences in retinal shape (41, 42). The reasons for the discrepancy between this study and our findings are unclear but may be related to differences in lens design or study population. Indeed, it has been demonstrated that the increases in AL over time is correlated with baseline refractive errors and age in patients wearing OK lenses, especially in high myopes (12, 26). In fact, lower myopes show a slower AL growth. The study by Santodomingo-Rubido et al. included significantly lower myopic patients in the OK group (mean myopia-2.10 ± 1.10D versus-3.1 ± 1.70D), and younger patients (9.6 ± 1.6 years versus 12.24 ± 2.49 years), which may have affected the impact of OK. Since younger subjects increases myopia faster, that may compensate the lower baseline myopia. Both factors may have affected the impact of OK efficacy in their study (40). Another possible explanation is the fact that our study was done with the latest generation orthokeratology contact lenses with customized optical zone designs than previous studies that typically used standard 6.00 mm of optic zone diameter. Latest studies with customized Back Optical Zone Diameter had found a CARE of 0.08 mm at 12 months in Caucasic and between 0.15 to 0.17 mm in Chinese children when compared DRL customized design with previous OK designs (29, 30, 43, 44).

Consistent with previous reports, this study found significant changes regarding all other biometrics parameters in the OK group (25, 39, 40, 45). The remodeling of the cornea by the OK lens resulted in a thinning of CCT of 9 microns and a shallower ACD related of an LT increase to compensate the decrease of the Anterior Segment Length (46). Hence, we did not observe any change in Anterior Segment Length over time. These findings confirm those of previous studies, indicating that the reduced axial elongation with OK was not overestimated by a decreased Anterior Segment Length and CCT (35, 47). Moreover, since AL is measured as the length between the tear layer and the inner limiting membrane the reduction of 9 microns of Corneal Thickness may represent as much roughly 0.01 mm of error in total AL measurement in OK sample not changing the results or conclusions of this study (48).

Several limitations were identified in the present study, mainly due to its retrospective design. Firstly, a selection bias may have been introduced, since the patients and their parents themselves chose the treatment modality. Secondly, the sample size of the present study was relatively small and the follow-up period was limited to 12 months. A larger-scale study with a longer follow-up period should be conducted to assess the long-term effect of OK on axial elongation. Thirdly, the wide range of ages within the sample may impact the final results, considering that myopia naturally slows down in older subjects. However, once compared by age and divided into two groups, lower age (7–13 years old) and higher age (14 to 17 years old) group, we found a lower increase in AL for the eldest compared to the younger glasses group (0.27 to 0.11 mm/year). Despite this, we found a 72% reduction in AL growth compared to controls in lower age group and 79% in higher age group. Expressed in absolute difference; 0.20 and 0.08 mm less AL grow at 12 month in young and eldest group, respectively. This indicates that the same ratio is maintained over time. In addition, greater effectiveness was found in the group of females once divided by sex.

The fact that refraction assessment was not performed under cycloplegia may also be regarded as a limitation and these results should be taken cautiously. However, all participants had previously undergone cycloplegia before entering the trial. In addition, the myopia progression was evaluated through the changes in AL. Therefore, this limitation is unlikely to have had a major impact on our main outcome measures based on AL eye growth.

In summary, this study provides further evidence that OK can effectively slow down myopia progression in children, with CARE of 0.14 ± 0.04 mm meaning 79% reduction in axial elongation compared to single vision-spectacles over a 12-month follow-up period. Notably, myopia progression was more pronounced in younger children, suggesting that strategies for myopia control should be initiated early even the efficacy of the treatment appears similar in both younger and older age groups.

## Data availability statement

The raw data supporting the conclusions of this article will be made available by the authors, without undue reservation.

## Ethics statement

The studies involving humans were approved by French Society of Ophthalmology Study published on the Health Data Hub n°F20220113001828. The studies were conducted in accordance with the local legislation and institutional requirements. Written informed consent for participation in this study was provided by the participants' legal guardians/next of kin. Written informed consent was obtained from the individual(s) for the publication of any potentially identifiable images or data included in this article.

## Author contributions

AQ: Formal analysis, Investigation, Validation, Writing – original draft. PR: Conceptualization, Data curation, Investigation, Methodology, Supervision, Writing – original draft, Writing – review & editing. KA-D: Conceptualization, Data curation, Investigation, Methodology, Writing – review & editing. J-PB: Conceptualization, Data curation, Investigation, Methodology, Writing – review & editing. J-BC: Conceptualization, Data curation, Investigation, Methodology, Writing – review & editing. AC: Conceptualization, Data curation, Funding acquisition, Investigation, Methodology, Validation, Visualization, Writing – review & editing. JP: Formal analysis, Funding acquisition, Investigation, Methodology, Supervision, Writing – original draft, Writing – review & editing.
